# Production and Purification of LTB-RBD: A Potential Antigen for Mucosal Vaccine Development against SARS-CoV-2

**DOI:** 10.3390/vaccines10101759

**Published:** 2022-10-20

**Authors:** Karla I. Solis-Andrade, Omar Gonzalez-Ortega, Dania O. Govea-Alonso, Mauricio Comas-Garcia, Sergio Rosales-Mendoza

**Affiliations:** 1 Facultad de Ciencias Químicas, Universidad Autónoma de San Luis Potosí, Av. Dr. Manuel Nava 6, San Luis Potosí 78210, Mexico; 2 Sección de Biotecnología, Centro de Investigación en Ciencias de la Salud y Biomedicina, Universidad Autónoma de San Luis Potosí, Av. Sierra Leona 550, Lomas 2ª. Sección, San Luis Potosí 78210, Mexico; 3 Sección de Microscopía de Alta Resolución, Centro de Investigación en Ciencias de la Salud y Biomedicina, Universidad Autónoma de San Luis Potosí, Av. Sierra Leona 550, Lomas 2ª. Sección, San Luis Potosí 78210, Mexico; 4 Sección de Genómica Médica, Centro de Investigación en Ciencias de la Salud y Biomedicina, Universidad Autónoma de San Luis Potosí, Av. Sierra Leona 550, Lomas 2ª. Sección, San Luis Potosí 78210, Mexico; 5 Facultad de Ciencias, Universidad Autónoma de San Luis Potosí, Av. Parque Chapultepec 1570, San Luis Potosí 78210, Mexico

**Keywords:** mucosal adjuvant, humoral response, chimeric antigen, mucosal vaccine, COVID-19

## Abstract

Most of the current SARS-CoV-2 vaccines are based on parenteral immunization targeting the S protein. Although protective, such vaccines could be optimized by inducing effective immune responses (neutralizing IgA responses) at the mucosal surfaces, allowing them to block the virus at the earliest stage of the infectious cycle. Herein a recombinant chimeric antigen called LTB-RBD is described, which comprises the B subunit of the heat-labile enterotoxin from *E. coli* and a segment of the RBD from SARS-CoV-2 (aa 439-504, carrying B and T cell epitopes) from the Wuhan sequence and the variant of concern (VOC)—delta. Since LTB is a mucosal adjuvant, targeting the GM1 receptor at the surface and facilitating antigen translocation to the submucosa, this candidate will help in designing mucosal vaccines (i.e., oral or intranasal formulations). LTB-RBD was produced in *E. coli* and purified to homogeneity by IMAC and IMAC-anionic exchange chromatography. The yields in terms of pure LTB-RBD were 1.2 mg per liter of culture for the Wuhan sequence and 3.5 mg per liter for the delta variant. The *E. coli*-made LTB-RBD induced seric IgG responses and IgA responses in the mouth and feces of mice when subcutaneously administered and intestinal and mouth IgA responses when administered nasally. The expression and purification protocols developed for LTB-RBD constitute a robust system to produce vaccine candidates against SARS-CoV-2 and its variants, offering a low-cost production system with no tags and with ease of adaptation to new variants. The *E. coli*-made LTB-RBD will be the basis for developing mucosal vaccine candidates capable of inducing sterilizing immunity against SARS-CoV-2.

## 1. Introduction

Coronaviruses are a group of positive-sense single-stranded RNA viruses with the largest and most stable known RNA genome. These viruses belong to the *Coronaviridae* family and are grouped into four genera: alpha-, beta-, gamma-, and delta-coronavirus. Of these four genera, only alpha- and beta-coronaviruses can infect humans. Most of the viruses that belong to these two genera can cause the “common cold” (e.g., CoV-NL63 and CoV-HKU1). However, three species can cause severe infections, leading to pneumonia and even resulting in death (i.e., SARS-CoV, SARS-CoV-2, and MERS-CoV). The appearance of SARS-CoV and MERS-CoV, which have a significantly higher mortality rate than SARS-CoV-2, led to the development of some vaccines. However, most of these efforts did not result in licensed vaccines, perhaps due to the limited human-to-human transmission. Furthermore, some SARS-CoV vaccines produced an antibody-dependent enhancement (ADE) infection in vaccinated individuals [1,2,3]. In the case of SARS-CoV, this phenomenon has been associated with vaccines displaying the full-length spike (S) protein. Most of the vaccines in clinical trials were either inactivated or live-attenuated viruses. Unfortunately, the high levels of human-to-human transmission for SARS-CoV-2 have resulted in a completely different scenario from the SARS-CoV and MERS-CoV epidemics. The scale and duration of the SARS-CoV-2 pandemic resulted in a worldwide halt of most activities. In some cases, these restrictions have been in place for a year, causing devastating and long-lasting effects that will take decades to overcome.

The COVID-19 pandemic has resulted in an extraordinary worldwide effort to develop novel vaccines quickly and safely against SARS-CoV-2 [4]. Interestingly, none of the approved vaccines has been based on the technologies used for SARS-CoV [5]. Instead, most of these vaccines are based on technologies that have been in the pipeline for quite a while [6]. The vaccines from Moderna/NIH and Pfizer/BioNTech rely on mRNAs encapsulated in lipids and polymers that code for the prefusion form of the spike (S) protein. In contrast, most of the remaining approved vaccines (i.e., AstraZeneca/Oxford, CanSino, Johnson & Johnson, and Gamaleya Institute) are based on adenoviral vectors [7,8,9]. The vaccine from Sinovac is formulated with the inactivated virus [10]. The efficacy of these vaccines varies between 60% and 98% [11]; however, the emergence of variants with mutations in the S protein (e.g., B.1.1.7, B.1.315, and P.1) could decrease the efficacy of these vaccines. Furthermore, the use of adenovirus-based vaccines (AdV5 and AdV26) has the drawback that countries with a high prevalence of these viruses can result in populations with neutralizing antibodies against them, further decreasing the efficacy of the vaccine [12,13]. Finally, one subunit-based vaccine (Novavax) is close to being approved in North America and Europe [14].

The production of biopharmaceuticals in *E. coli* offers a robust and well-established platform that can be easily transferred from an academic laboratory to a manufacturing facility [15]. This expression system can be easily scaled up, and the regulatory aspects of the production have been in place for a long time such that they are considered standard. Recombinant subunit vaccines have the advantages of not containing a pathogenic organism (viral or bacterial), their composition is exactly known for each batch, they can be produced using different platforms and fermentation processes, large-scale production is relatively simple and cost-effective, and both the expression system and the antigen can be easily modified by genetic engineering [16,17,18]. It is important to point out that contrary to live-attenuated, inactivated, and viral vector-based vaccines, the level of biosafety required for the expression of recombinant subunit vaccines is lower; therefore, their overall cost can be significantly lower. Furthermore, there is no risk of the vaccine resulting in a viral infection due to defective manufacturing procedures. Subunit vaccines still must overcome some challenges. In general, multiple doses are required due to their low immunogenicity compared to the use of the whole pathogen. One approach to enhance the immunogenicity consists in fusing (genetic or chemically) the antigen of interest with a highly immunogenic protein subunit from bacteria (e.g., the *E. coli* heat-labile enterotoxin B subunit toxin [LTB] [19,20] or cholera toxin B subunit [CTB]). A major challenge when expressing recombinant proteins in bacteria is to obtain the antigen in the properly folded state, i.e., to avoid the expressed protein from generating inclusion bodies, which requires protein refolding to its native and functional state. This problem can be solved by fusing the protein with another protein that increases antigen solubility inside the cell (e.g., SUMO) [21], although this protein must be cleaved from the antigen once it is in a stable form and purified. Nonetheless, a rational protein design that takes into consideration the fermentation and purification protocols, the secondary and tertiary structure of the target antigen, the amount and distribution of hydrophobic amino acids, and the sequences that can be recognized by the bacterium can greatly increase the solubility of the antigen in the cell [22,23,24].

Considering that the current COVID-19 vaccines are intramuscularly administered and that the lack of prevention of virus infection is associated with a poor induction of mucosal IgA responses, there is an urgent need to explore new vaccination approaches focused on the induction of effective mucosal responses. One straightforward approach to achieving this goal is the development of nasal or oral vaccines, which effectively boost the mucosal immune system in the compartment used for vaccine delivery and even in distant compartments. It is well known that oral immunization results in GALT-mediated antigen processing with subsequent homing to pulmonary tissues in which IgA production is induced [25]. In the current scenario, such mucosal vaccines may serve as boosters of the immunity induced by parenterally-administered vaccines.

Since the B subunit of *E. coli* enterotoxin is recognized as a potent mucosal adjuvant, in this study a chimeric protein (LTB-RBD) based on this carrier and a segment of RBD targeting T and B cell epitopes is reported. The production in *E. coli* of LTB-RBD and its purification was performed, and evidence of the immunogenic potential by parenteral and mucosal routes of this chimeric protein was generated.

## 2. Materials and Methods

### 2.1. Expression Vector Design

Two synthetic genes coding for a chimeric protein called LTB-RBD Wuhan strain and VOC delta, respectively, were obtained by GenScript Inc. (Piscataway, NJ, USA), following a codon optimization process according to codon usage in *E. coli*. The sequence comprises the full-length sequence of LTB fused to aa 439–504 from the S protein. A GPGP linker was placed between the LTB and RBD moieties to facilitate displaying the target antigen. The structure of the recombinant proteins was modeled using the Phyre2 protein fold recognition server [26]. NdeI and XhoI restriction sites were placed at the 5′and 3′ ends, respectively, to facilitate subcloning into pET 21b (+), in which the ORF is fused to a His tag coding sequence at the 3′ end in the case of the Wuhan strain, and without His tag in the case of VOC delta. These procedures were performed following standard molecular cloning protocols. A positive clone carrying the target expression vector was confirmed by restriction profiling and conventional sequencing.

### 2.2. Strains, Materials, and Culture Media

The pET 21b (+)-LTB-RBD construct was transferred to the *E. coli* Rosetta (DE3) pLysS host. The transformation was performed by heat shock. Afterward, the cells were shaken at 37 °C for 1 h and streaked on LB plates supplemented with ampicillin (100 mg/L) and chloramphenicol (40 mg/L). Cultivation conditions in shake flask cultures were as follows: a single positive colony was inoculated in 500 mL baffled flasks containing 100 mL of LB medium (10 g/L bacto-peptone, 5 g/L yeast extract, and 10 g/L NaCl) supplemented with ampicillin (100 mg/L) and chloramphenicol (40 mg/mL) at 37 °C and 140 rpm. To induce expression of the LTB-RBD fusion protein, cells were grown to an optical density of 0.7–1.0 at 600 nm (OD_600 nm_), and lactose (15 g/L) or IPTG (0.1 mM) was added. All shake flask cultures were induced at 28 °C. Induction was maintained for 7 h, and samples were withdrawn at 0, 4, and 7 h. Cultivation conditions in batch bioreactor cultures were as follows. A seed culture was prepared in a 1 L flask containing 200 mL of LB medium, incubated at 37 °C, and 140 rpm for 16 h. The cells were harvested by centrifugation and resuspended in 20 mL of fresh LB medium. Upon inoculation of the bioreactor, an OD_600 nm_ of 0.4–0.5 was reached. Batch cultures were grown in a 1.5 L jar fermenter (ez-Control system model 56,156, Applikon Biotechnology, Delft, The Netherlands) containing 1 L of LB medium plus ampicillin (100 mg/L) and chloramphenicol (40 mg/L). pH was maintained at 7.0 ± 0.5 by adding 2 M HCl or 2 M NaOH, and O_2_ saturation was kept above 40% by culture stirring (400–600 rpm) and aeration (0.5–1.5 L/min). The temperature was held at 37 °C. When culture density reached an OD_600 nm_ of 1.0–1.5, the temperature was decreased to 28 °C, and lactose was added to reach a concentration of 15 g/L and induce expression of LTB-RBD. The batch bioreactor culture was induced for 9–12 h, and 10 mL samples were collected at 3 h intervals. The samples and the endpoint cultures, either from flask or bioreactor fermentations, were centrifuged at 6000 rpm for 10 min at 4 °C, and the pellets were stored at −40 °C. The induced and noninduced samples were analyzed by sodium dodecyl sulfate–polyacrylamide gel electrophoresis (SDS-PAGE) and/or Western blotting.

### 2.3. Cell Disruption

The bacterial biomass was collected at 7000 rpm (5 min) and resuspended in a cold solution (100 mM Tris-HCl, 20% (*w*/*v*) sucrose, and pH 7.4). 0.2 mL of this solution was used per 20 mg of biomass. The cells were recovered by centrifugation at 7000 rpm for 15 min (4 °C). The pellet was resuspended in injectable water + 0.01 mM PMSF (0.5 mL per 20 mg of biomass). Cells were disrupted while the test tube was kept on ice by applying 6–9 cycles of 30 s on and 30 s off using ultrasonication (GEX130PB device, Twinsburg, OH, USA) at a 70% amplitude. Afterward, the suspension was centrifuged at 7000 rpm for 30 min (4 °C) to recover a pellet composed of inclusion bodies and insoluble cellular components and a supernatant (soluble protein fraction). These fractions were stored at −40 °C until further analysis.

### 2.4. SDS-PAGE and Western Blotting Analyses

Protein samples were mixed with 5× reducing dye buffer (500 mM DTT, 250 mM Tris-HCl, 10% (*w*/*v*) SDS, 50% (*v*/*v*) glycerol, 0.1% bromophenol blue, pH 6.8) and boiled for 10 min. Proteins were separated using a 12% denaturing polyacrylamide gel and visualized by Coomassie blue staining. For Western blot analysis, the proteins were transferred from the polyacrylamide gel to a nitrocellulose membrane (Thermo Fisher Scientific, Waltham, MA, USA) for 1 h at 500 mA using an electrophoretic transfer cell (Biorad, Hercules, CA, USA). The membrane was incubated overnight in blocking buffer (5% (*w*/*v*) fat-free dry milk dissolved in PBS + 0.05% (*v*/*v*) Tween 20). The membrane was subsequently washed three times with PBS + 0.05% (*v*/*v*) Tween 20 and incubated with mouse anti-sera (1:1000 dilution) against either the cholera toxin B subunit (CTB), which is an in-house obtained mouse hyperimmune serum using complete Freund’s adjuvant and commercial CTB from Sigma (cat. no. C9903), or a monoclonal mouse antibody (1:3000 dilution) targeting the His tag. The blots were washed and incubated with a goat horseradish peroxidase-conjugated secondary anti-IgG mouse antibody (1:2000 dilution, Sigma, Livonia, MI, USA) for 2 h at room temperature. Antigen detection was performed by incubating blots with the SuperSignal West Pico chemiluminescent substrate (Pierce, Rockford, IL, USA), and the signal was revealed using chemiluminescent-sensitive Kodak film (Kodak, Rochester, NY, USA).

### 2.5. Analytical Methods

During flask and batch cultivation, cell growth was monitored by measuring OD_600 nm_. Protein samples were quantified with a protein Bradford assay kit (Ab 102535, Abcam, Cambridge, UK) using bovine serum albumin as standard.

### 2.6. Protein Purification

The insoluble fraction obtained upon cell disruption was subjected to a washing procedure (twice with PBS 1× + 1% (*v*/*v*) Triton X-100 and twice with PBS 1×) to remove cellular components and solubilize contaminant proteins. The washed pellet was contacted with solubilizing buffer (20 mM phosphate, 500 mM NaCl, 8 M urea, pH 7.1) overnight at 4 °C. Finally, the suspension was centrifuged at 13,000 rpm for 20 min to recover the supernatant with solubilized recombinant protein. This step was repeated twice. This supernatant is the sample used for chromatography.

For the LTB-RBD Wuhan sequence construct, which carries a His tag, IMAC was run using a 2 mL column packed with Chelating Sepharose Fast Flow (Pharmacia Biotechnology, Stockholm, Sweden). The adsorbent was charged with Ni^2+^ ions and equilibrated with binding buffer, and 1 mL of the previously obtained protein extract was injected to the chromatographic system at a flow rate of 0.25 mL/min. After washing the column, protein desorption was accomplished by feeding the desorption buffer (20 mM phosphate, 500 mM NaCl, 500 mM imidazole, 8 M urea, pH 7.1). Fractions containing the protein of interest were collected and subjected to a refolding process using dialysis with a 6–8 kDa MWCO membrane. The buffers used to accomplish refolding, in sequential order, were 20 mM phosphate buffer + 4 M urea at pH 7.1, 50 mM carbonate + 10% (*v*/*v*) glycerol + 0.01% (*v*/*v*) Tween at pH 9.2, and 10% (*w*/*v*) sucrose + 0.01% (*v*/*v*) Tween 20.

For the LTB-RBD delta variant construct, which lacks tags, the supernatant with solubilized protein was used for IMAC as described above; however, in this case, the fractions containing the protein of interest were collected as unbound protein (several contaminant proteins bound to the immobilized Ni^2+^ ions). After this purification step, a change in buffer (20 mM Tris-HCl, 10 mM NaCl, 8 M urea, pH 8.6) was applied using dialysis to reduce the concentration of NaCl. A second purification step using anionic exchange chromatography was performed. The fractions obtained containing the recombinant protein were harvested again as unbound protein (the few contaminant proteins found after IMAC were retained by the cationic column) and dialyzed using the following buffers sequence: 20 mM phosphate buffer + 4 M urea at pH 7.1, 50 mM carbonate + 10% (*v*/*v*) glycerol + 0.01% (*v*/*v*) Tween at pH 9.2, and 10% (*w*/*v*) sucrose + 0.01% (*v*/*v*) Tween 20.

### 2.7. Protein Concentration

The purified protein was concentrated using powdered polyethylene glycol (PEG, MW 200,000, Sigma) or by ultrafiltration. The protein solution was transferred to a 6–8 kDa MWCO dialysis membrane, which was covered with PEG powder. After a 20 min incubation at 4 °C, the layer of hydrated PEG over the dialysis bag was removed and replaced with dry PEG. This procedure was repeated 3–5 times. As for ultrafiltration, a Vivaspin 2 column (5 kDa MWCO) was rinsed once by adding a solution composed of 10% (*w*/*v*) sucrose + 0.01% (*v*/*v*) Tween 20 and centrifuged at 4000 rpm by 10 min. Afterward, the protein solution was placed into the column and concentrated by two cycles of centrifugation of 30 min at 4000 rpm. After these steps, the concentrated protein was quantified.

### 2.8. Immunogenicity Assessment

The immunogenicity of LTB-RBD was assessed in BALB/c mice (n = 4, 12 weeks old), following a protocol approved by the institutional ethics committee (CEID-2020-07R1). The groups received one of the following treatments on days 1 and 14: 5 µg of LTB-RBD plus alum by subcutaneous (s.c.) route, 10 µg of LTB-RBD plus alum by s.c. route, 10 µg of LTB-RBD alone by oral (p.o.) route, 10 µg of LTB-RBD plus 1 µg of cholera toxin, 3 µg of LTB-RBD alone by intranasal (i.n.) route, or 3 µg of LTB-RBD plus 0.3 µg of cholera toxin by i.n. route. Negative control groups were treated with the antigen vehicle alone (10% sucrose, 0.01% Tween 20) by s.c., p.o., or i.n. routes. Dose volumes were the following: 300 µL for s.c., 400 µL for p.o., and 20 µL for i.n. routes. For s.c. formulations, Alum adjuvant was used at a 1:5 ratio (G Biosciences, St. Louis, MO, USA, cat no. 786-1215). Animals were slightly anesthetized with isoflurane right before immunization.

All samples were taken from all mice groups on day 27. Blood samples were withdrawn by puncture in the tail. After clot formation, samples were centrifuged at 10,000 rpm for 10 min, and the obtained sera were stored at −20 °C until antibody determination. Feces and mouth wash samples were taken as follows. For feces, 100 mg was collected and resuspended in 500 μL of ice-cold PBS (supplemented with 5% fat-free dry milk and 1 mM of PMSF). Following homogenization using a plastic device, the samples were centrifuged at 7000 rpm and 4 °C for 15 min. Supernatants were transferred to a new tube and kept at 4 °C for immediate analysis by ELISA. Mouth-wash samples were obtained from mice anesthetized with isoflurane, which were subjected to mouth wash with 120 μL of PBS. The obtained washes had 1 mM PMSF added. Feces extracts and mouth-wash samples were immediately plated for antibody determination by ELISA.

ELISA was run to measure S protein binding antibodies following previously reported protocols [27]. Polystyrene plates (96 wells) were coated overnight at 4 °C with spike protein (100 ng/well, Sinobiological cat. no. 40589-V08H4) in carbonate buffer (15 mM Na_2_CO_3_, 35 mM NaHCO_3_, pH 9.6). Before all following steps, plates were washed three times with PBS + Tween 0.05% (PBS-T). Plates were blocked with 5% fat-free dry milk in PBS for 2 h at room temperature. Serial dilutions of sera (1:40–1:160) or mouth wash and fecal extracts (1:1 and 1:2) in PBS were added and incubated at 4 °C overnight. Afterward, for all samples, secondary antibodies labeled with goat horseradish peroxidase-conjugated anti-mouse IgG for sera samples or anti-mouse IgA for feces and mouth-wash samples were diluted in PBS (dilution 1:2000) and plated. Finally, an ABTS substrate solution [0.6 mM 2,20-azino-bis(3-ethylbenzothiazoline-6-sulfonic acid) + 0.1 M citric acid + 1 mM H_2_O_2_, pH 4.35] was added and OD values at 405 nm were measured after 30 min using a MultiskanR FC equipment (Thermo Scientific, Waltham, MA, USA). IgG antibody titers were determined as the reciprocal of the highest dilution of sera with a mean OD value above the OD value from the group treated with the vehicle alone (negative control) plus 2 × SD. Statistical differences were determined using one-way ANOVA employing the GraphPad Prism version 5.01 software (*p* < 0.05).

## 3. Results

**Figure 1:**
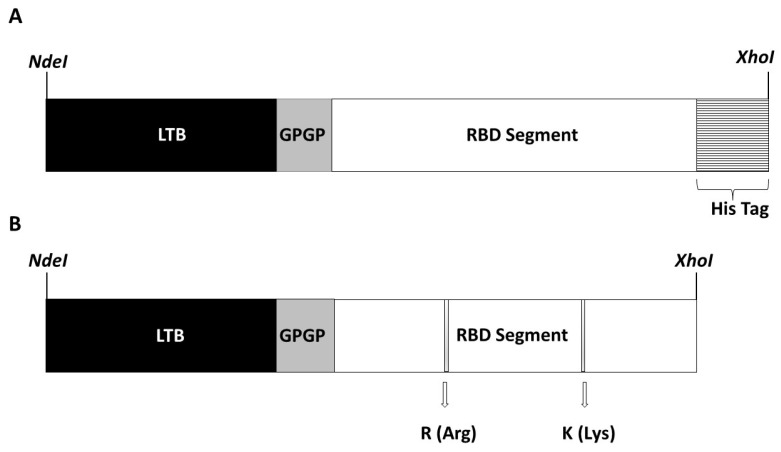
Schematic representation of the chimeric proteins produced and evaluated in this study. (**A**) Map of the chimeric protein LTB-RBD targeting the Wuhan isolate. (**B**) Map of the chimeric protein LTB-RBD targeting the delta variant. The fusion proteins comprise the full-length sequence of LTB (in black), a GPGP linker (in gray), a segment of the RBD from SARS-CoV-2 (aa 439–504, carrying B and T cell epitopes, in white), and hexa-histidine tag (in horizontal stripes). The delta variant construct has two amino acid changes (arrows): arginine (R) instead of leucine (L) and lysine (K) instead threonine (T). *Nde*I and *Xho*I restriction sites were placed at the 5′ and 3′ ends, respectively, to facilitate subcloning into the pET 21b (+) vector.

**Figure 2:**
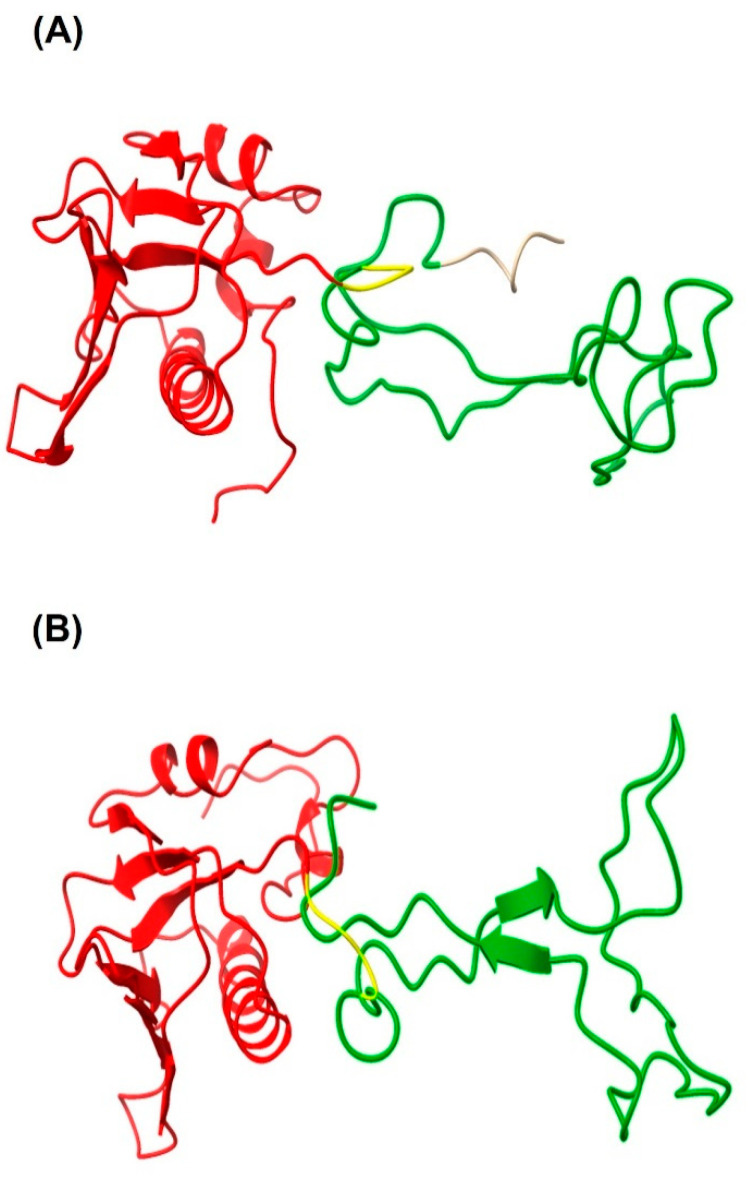
A 3D structure model of the candidate vaccine LTB-RBD targeting the Wuhan sequence (**A**) or delta variant (**B**). The full-length sequence of LTB (red) fused to an RBD segment (aa 439–504, green). GPGP linker (yellow) placed between the LTB and RBD domains. The His tag is only present in LTB-RBD Wuhan. These structures were modeled with the Phyre2 server.

**Figure 3:**
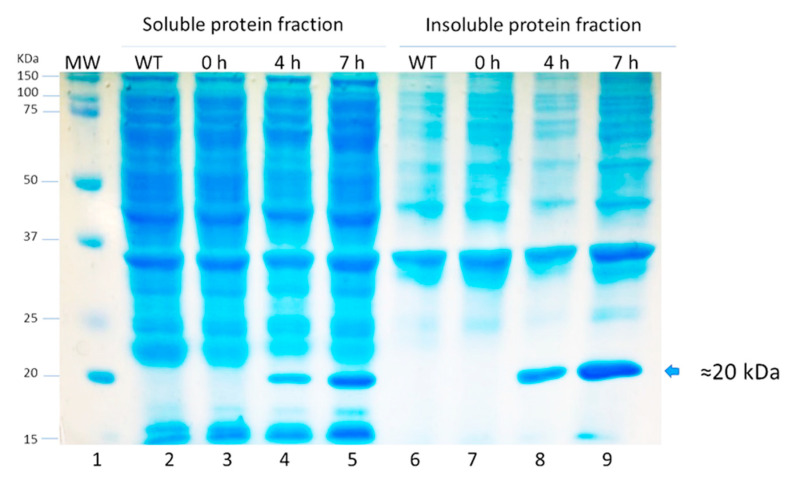
SDS-PAGE analysis of protein extracts from cultures expressing LTB-RBD Wuhan induced for 4 and 7 h at 28 °C with lactose (lanes 2 to 5: soluble protein fraction and lanes 6 to 9: insoluble protein fraction). Lane 1: molecular weight marker, lanes 2 and 6: untransformed cultures, lanes 3 and 7: uninduced cultures, lanes 4 and 8: cultures induced for 4 h, and lanes 5 and 9: cultures induced for 7 h.

The chimeric protein LTB-RBD is based on the LTB carrier and a segment of RBD (aa 439–504 from the S protein). The sequence was chosen because the RBD is considered a crucial antigen targeting T and B cell epitopes. In this study, two constructions were explored based on the RBD of the Wuhan sequence and VOC delta. The main difference between the delta variant and Wuhan sequences is two amino acid changes, arginine (R) instead of leucine (L) and lysine (K) instead of threonine (T). The sequences were modeled using the Phyre2 engine to determine the secondary structure. The in silico analysis did not predict radical changes in the secondary structure between the Wuhan sequence and delta variant due to these amino acid changes (Figure 1 and Figure 2). The expression of LTB-RBD in a Rosetta-pET21b (+)-flask system was assessed using 0.1 mM IPTG at two induction times (4 and 7 h). Analysis of the SDS-PAGE results revealed the presence of a 21 kDa protein in the cultures of both variants induced that was absent in both the preinduction cultures and WT cultures, matching with the theoretical molecular weight for the mature form of LTB-RBD Wuhan sequence (20.5 kDa) since the protein MW comprising the signal peptide is 22.9 kDa (Figure 3). The recombinant LTB-RBD was detected in both the soluble and insoluble fractions with similar abundance in such fractions. As for the LTB-RBD delta construct, similar findings were obtained in terms of expression in inclusion bodies and recombinant protein yields in crude extracts (data not shown). However, in this case, no soluble recombinant protein was observed.

**Figure 4:**
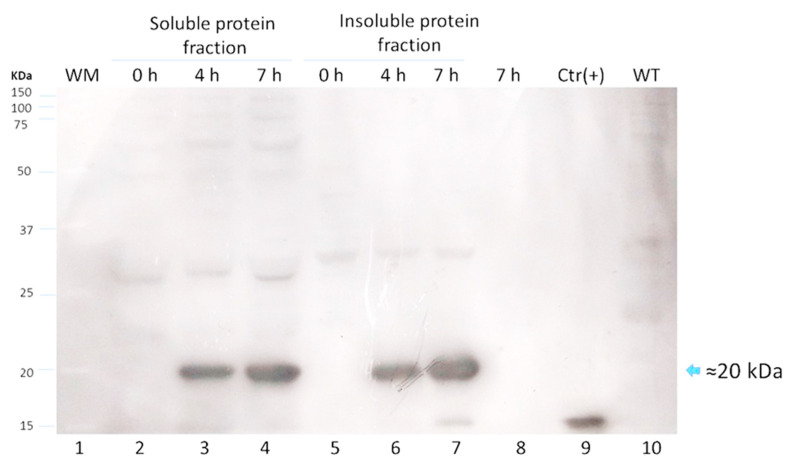
Western blot analysis for LTB-RBD Wuhan using anti-cholera toxin B subunit (CTB) serum to analyze protein extracts from cultures induced for 4 or 7 h at 28 °C with lactose as inducer (lanes 2 to 4: soluble protein fraction and lanes 5 to 7: insoluble protein fraction). Lane 1: molecular weight marker, lanes 2 and 5: uninduced cultures, lanes 3 to 6: cultures induced for 4 h, lanes 4 and 7: cultures induced for 7 h, lane 8: culture medium supernatant, lane 9: cholera toxin subunit (CTB) used as positive control, and lane 10: soluble fraction of an untransformed culture.

**Figure 5:**
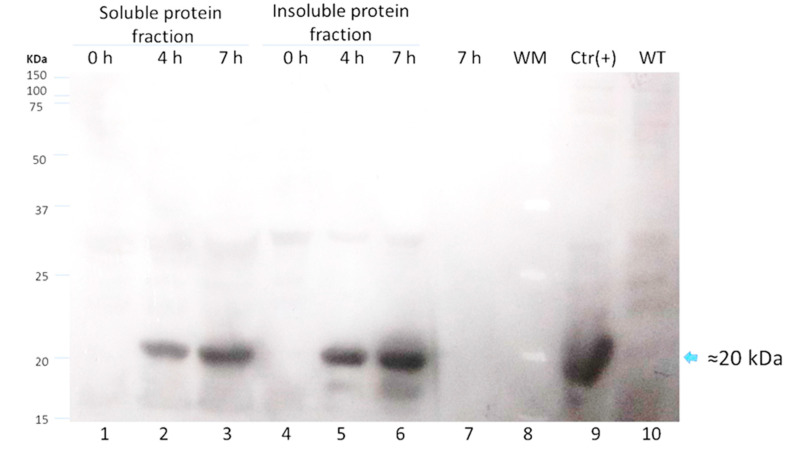
Western blot analysis for LTB-RBD Wuhan using anti-His tag of protein extracts from cultures induced for 4 or 7 h at 28 °C with lactose as inducer (lanes 1 to 3: soluble protein fraction and lanes 4 to 6: insoluble protein fraction). Lanes 1 and 4: uninduced cultures, lanes 2 and 5: cultures induced for 4 h, lanes 3 and 6: cultures induced for 7 h, lane 7: culture medium supernatant, lane 8: molecular weight marker, lane 9: RBD protein as positive control, and lane 10: soluble fraction of an untransformed culture.

Since lactose is a more convenient inducer (lower cost and toxicity) than IPTG, the production of LTB-RBD was assessed using 15 g/L lactose as inducer [28]. SDS-PAGE analysis revealed that the target protein was expressed roughly at similar levels with no major variation in solubility (Figure 3). To further confirm the antigenicity of the *E. coli*-produced LTB-RBD, a Western blot was performed using either an anti-His antibody or an anti-CTB hyperimmune serum. All these analyses revealed an immunoreactive protein of the same molecular weight that matched the differential protein detected in the SDS-PAGE analysis, confirming the identity and antigenic activity of both LTB and RBD sequences (Figure 4 and Figure 5).

**Figure 6:**
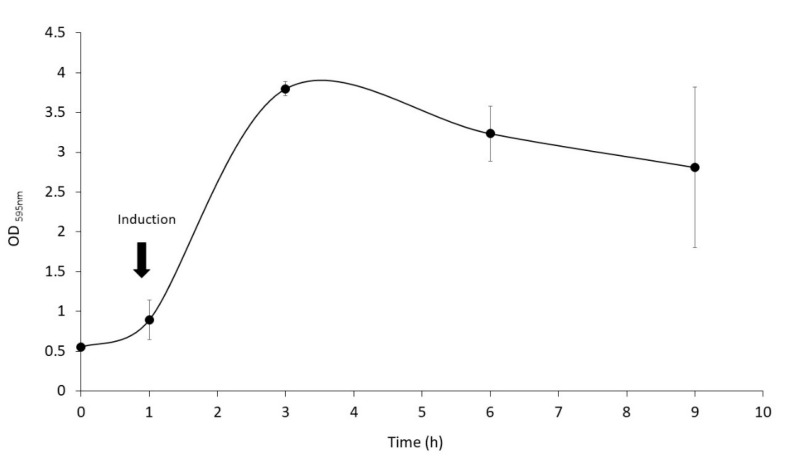
Kinetics for the fermentation in a 1.5 L bioreactor. The LTB-RBD Wuhan antigen was produced in batch cultures grown in a 1.5 L jar fermenter (ez-Control system model 56,156, Applikon Biotechnology) containing 1 L of LB medium plus antibiotics. pH was maintained at 7.0 ± 0.5, O_2_ saturation was kept above 40% by culture stirring (400–600 rpm) and aeration (0.5–1.5 L/min). The temperature was held at 37 °C.

**Figure 7:**
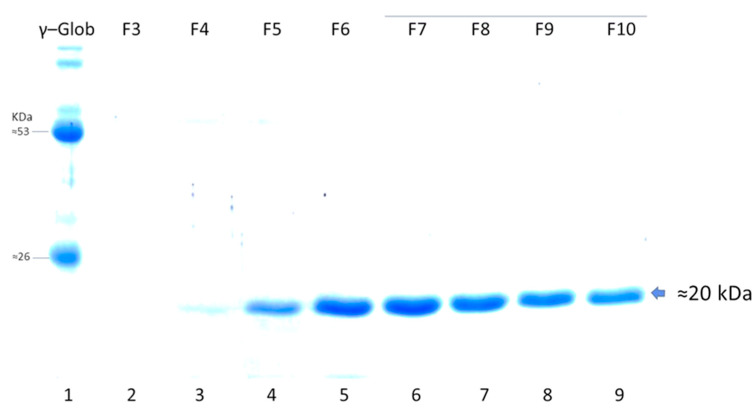
SDS-PAGE analysis of elution fractions during IMAC purification of LTB-RBD Wuhan. Lane 1: γ globulin from human blood as molecular weight marker, lanes 2 to 9: eluted fractions upon entrance of imidazole for protein desorption.

**Figure 8:**
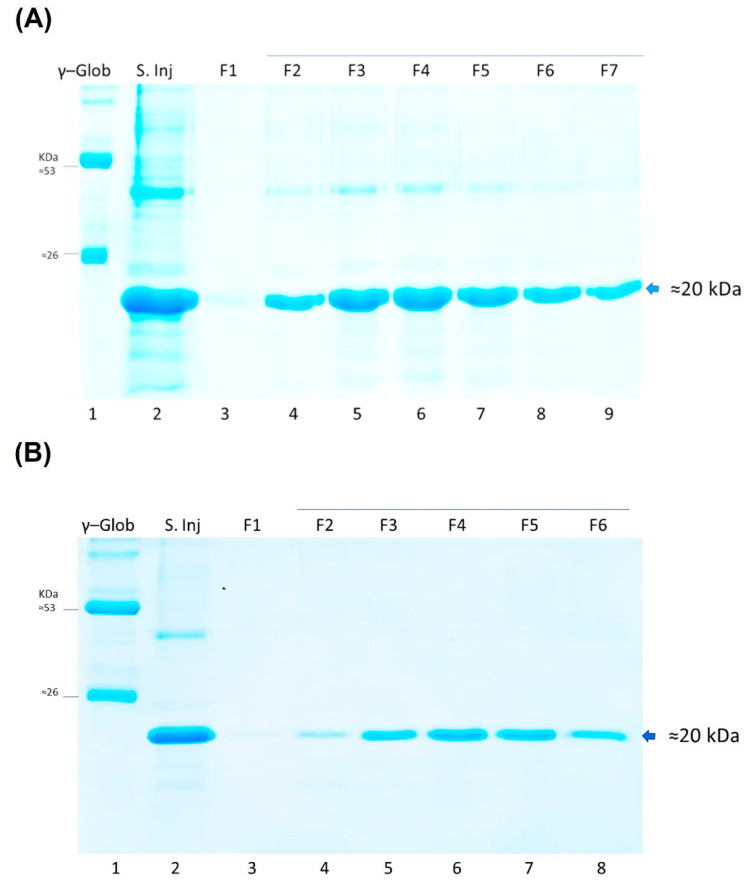
(**A**) SDS-PAGE analysis of eluted fractions during IMAC purification of LTB-RBD delta variant. Lane 1: γ globulin from human blood as molecular weight marker, lane 2: sample injected into IMAC column, and lanes 3 to 9: eluted fractions upon entrance of imidazole for protein desorption. (**B**) SDS-PAGE analysis of elution fractions during anionic exchange chromatography. Lane 1: γ globulin from human blood as molecular weight marker, lane 2: sample injected into anionic column, and lanes 3 to 8: eluted fractions upon entrance of NaCl for protein desorption.

**Figure 9:**
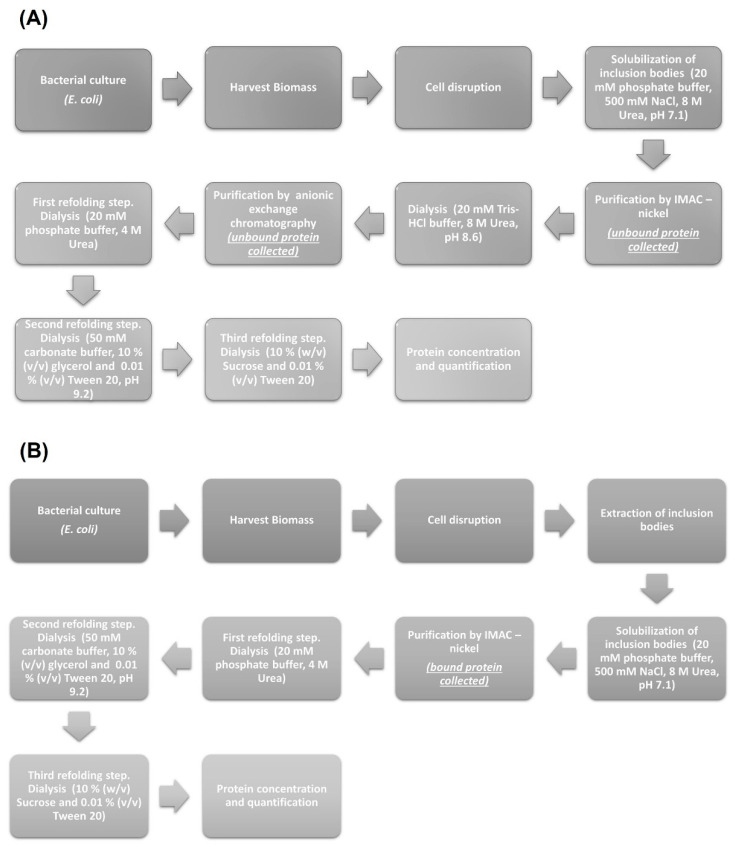
Description of the steps followed in the methodology implemented for the purification of LTB-RBD antigens. (**A**) Protein purification workflow for LTB-RBD Wuhan, which has a His tag. (**B**) Protein purification workflow for LTB-RBD delta, which lacks tags.

The production of LTB-RBD was further scaled up to a 1 L bioreactor using lactose as inducer. The analysis of biomass fractions withdrawn over a period of 9 h in the postinduction phase revealed that the recombinant protein accumulation peaked at 6 h postinduction and remained at a similar level at the end time point (9 h postinduction, Figure 6). In terms of solubility, a shift was observed with respect to flask fermentations, with an increase in the abundance of the protein in the insoluble fraction. The biomass showed a constant increase with a maximum density of 7.5 g/L (OD_600nm_ = 3.0) reached at the endpoint (Figure 6). The purification method based on IMAC allowed obtaining pure LTB-RBD Wuhan with average yields of 1.2 mg per liter of culture (Figure 7). As for the LTB-RBD delta construct, the purification method based on IMAC followed by anionic exchange chromatography allowed reaching yields of 3.5 mg of pure LTB-RBD per liter of culture (1.4 mg of protein per g of fresh biomass, Figure 8). In Figure 9, a summary of the implemented protocols for the two versions of the LTB-RBD produced in this report is presented.

**Figure 10:**
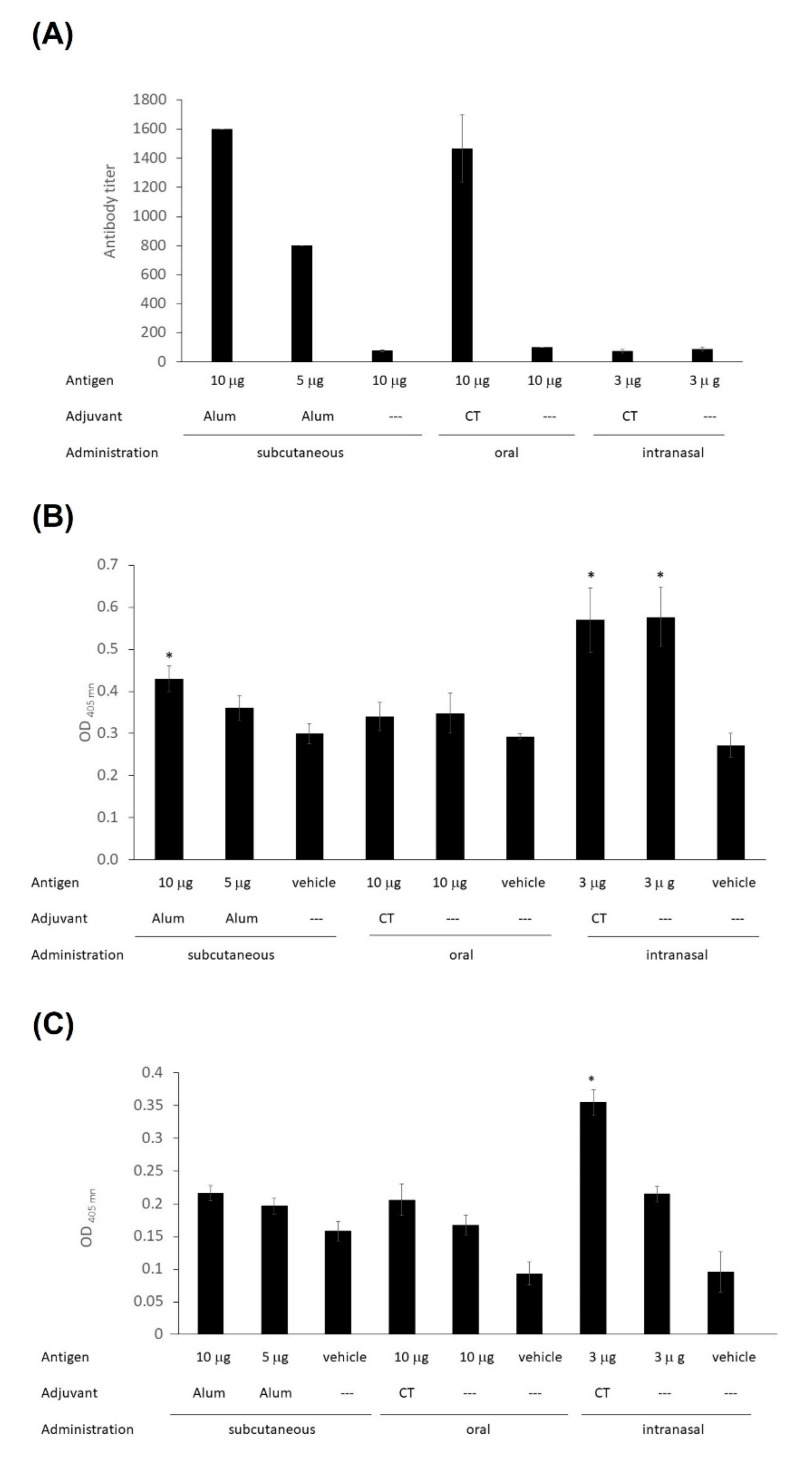
Humoral response induced by LTB-RBD in test mice. (**A**) IgG titers measured in serum. (**B**) IgA levels measured in mouth washes. (**C**) IgA levels measured in feces. Antibodies were measured by ELISA using the S protein as the target antigen. Samples were collected on day 27. The error bars are the standard deviation of the mean absorbance value. Asterisks denote significant differences (*p* < 0.05) versus the control group (vehicle).

The immunogenicity of LTB-RBD was assessed in test mice subjected to two vaccine doses administered by distinct routes. The assessment of anti-spike IgG levels revealed that significant IgG responses were induced in the s.c. immunized group, with a higher response in the high-dose group (average titers of 1600 for the 10 μg LTB-RBD + Al(OH)_3_ group and 800 for the 5 μg LTB-RBD + Al(OH)_3_ group). Interestingly, the group orally immunized with 10 μg LTB-RBD + CT had a titer value of 1600, whereas the formulation lacking the CT adjuvant showed very low antibody titer. In contrast, nasal immunization failed to induce relevant IgG responses, regardless of the use of CT as adjuvant (Figure 10A).

Regarding the mucosal immune response, IgA measurements in saliva (mouth washes) revealed that significant levels were triggered in the groups immunized by the nasal route, regardless of the use of CT as adjuvant and in the s.c. immunized group receiving the high antigen dose (Figure 10B). Intestinal IgA responses were evaluated by measuring IgA levels in feces, showing that only the nasally immunized groups triggered a significant response, regardless of the use of CT as adjuvant (Figure 10C).

## 4. Discussion

In the present study, LTB-RBD (a chimeric protein comprising a mucosal adjuvant carrier (LTB) and a segment from the RBD of SARS-CoV-2) was produced and purified as an antigen vaccine candidate for the formulation of mucosal vaccines against COVID-19.

The LTB-RBD candidate was expressed as inclusion bodies, which is in line with the report by Jegouic et al., who assessed the expression of fragments of the S protein from SARS-CoV-2, including segments covering the RBD sequence; however, they remained in inclusion bodies despite performing the expression at a lower temperature (15 °C) and with different IPTG concentrations [29]. Moreover, recombinant LTB has been previously reported to be mainly produced as inclusion bodies in *E. coli* [30,31]. The LTB-RBD antigen accumulated as inclusion bodies was subjected to purification and refolding procedures. The presence of urea in the solubilization procedure allowed purifying the candidate using IMAC with Ni^2+^ ions. In-column refolding was tried, but the results were poor since the protein precipitated inside the column upon urea removal. Once the candidate was desorbed from the Ni^2+^ ions using imidazole, the eluted fractions containing highly pure protein were subjected to refolding using dialysis. Several buffers were tested to accomplish refolding: the sequence of buffers that successfully produced refolded protein was PBS + 4 M urea at pH 7.1, 50 mM carbonate + 10% (*v*/*v*) glycerol 0.01% (*v*/*v*) + Tween 20 at pH 9.2, and 10% (*w*/*v*) sucrose + 0.01% (*v*/*v*) Tween 20 (Table 1). The use of PBS as final buffer for the candidate was forbidden, as it completely precipitated the candidate upon freezing.

Studies performed with SARS-CoV sequences revealed that the RBD produced in *E. coli* is antigenic and immunogenic, although at a lower magnitude than that expressed in mammalian cells, while no yields were reported for the *E. coli* system [32]. RBD has also been expressed fused to a solubility-enhancing peptide (SEP) tag containing nine arginine residues, resulting in an enhancement in the accumulation of soluble RBD. This RBD version was produced at yields of up to 2 mg/L and recognized the ACE2 receptor and induced antibodies able to interact with a mammalian made S1 protein [33]. Another case is the report by McGuire et al. [34], in which fusion proteins were designed based on the thermophilic family of nine carbohydrate-binding modules (CBM9) as an N-terminal carrier protein and affinity tag and different S protein segments. Among the proteins tested, the one called CBM9-ID-H1 carrying amino acids 540–588 from the S protein was produced at yields up to 122 mg/L of pure protein, which was widely reactive with COVID-19 convalescent sera, suggesting that it retains the antigenic determinants; therefore, it is proposed as a promising immunogen. These studies support the use of *E. coli* to produce functional SARS-CoV-2 antigens, a system that offers lower cost compared to mammalian cell-based platforms.

The functional activity of LTB-RBD was initially assessed in mice subjected to two immunizations by different routes (subcutaneous, oral, and nasal). Since sera from mice s.c. receiving the LTB-RBD antigen (10 µg) plus alum as adjuvant showed significant serum anti-S IgG responses (and modest anti-S IgA responses in the mouth), we believed that this antigen has a promising potential to induce SARS-CoV-2 neutralizing antibodies. Since LTB-RBD was able to induce both systemic IgG responses and modest IgA responses in mucosal compartments when s.c. administered but induced poor seric IgG levels and high IgA responses when i.n. administered, we propose that combining the administration routes in the immunization scheme (i.e., a third boosting i.n. dose in the s.c. immunized group) might lead to optimal immune response comprising robust humoral responses in both the systemic and mucosal levels. These findings justify expanding the characterization of LTB-RBD to assess the cellular response and perform neutralization assays to determine the immunoprotective potential of these vaccine candidates. Currently, seven intranasal anti-SARS-CoV-2 vaccines have reached clinical evaluation. Most of these developments are based on viral vectors or live attenuated viruses [35]. The oral delivery of SARS-CoV-2 vaccines has also been explored. For instance, Jawalagatti et al. [36] reported a Salmonella strain delivering a replicon coding for SARS-CoV-2 RBD, HR, M, and epitopes of nsp13 (RNA helicase), which successfully induced protective immunity in mouse and hamster models of SARS-CoV-2 infection associated to cellular and humoral responses, including the efficient induction of IgA responses in the respiratory mucosa.

Immunogenic carriers are essential when the target antigen has low complexity and is therefore not very immunogenic. LTB is a promising carrier as it has immunomodulatory effects and facilitates the translocation of the fused antigen into the submucosa, in addition to increasing the complexity of the antigen. LTB has been used experimentally in numerous studies as carrier of unrelated antigens, with the ability to favor the induction of humoral responses and memory B lymphocytes. LTB enhances the humoral response against unrelated, genetically fused antigens when administered by either i.n. or oral routes [37,38]. In contrast to the LT holotoxin, LTB is not an inherently toxic protein and has been used as adjuvant in a vaccine candidate against ETEC diarrhea that reached clinical trials with positive results [39]. Moreover, LTB has also proven adjuvant activity when parenterally administered [40]; therefore, the design of combined parenteral–mucosal immunization scheme could be achieved with the LTB-RBD antigen to induce proper immune responses in terms of potency and compartmentalization (i.e., parenteral priming and mucosal boosting), offering the potential to prevent virus spread at early stages of infection. Interestingly, sublingual immunization has also been proposed as a convenient route able to induce high immune responses and deserves further exploration for the LTB-RBD antigen [41]. LTB has been used as antigen/adjuvant in an oral vaccine against enterotoxigenic *E. coli*; it induced antibodies and memory B lymphocytes with no serious adverse effects [42]. Based on this background, LTB was chosen as the carrier agent that will hypothetically increase the induction of immune responses toward SARS-CoV-2 epitopes after administration of the antigen by the nasal or oral route. In this respect, the development of mucosal vaccines not only represents an attractive advantage in terms of friendlier administration (more acceptable by patients) but also the opportunity to achieve the induction of more attractive immunological profiles, considering that immunization by these routes leads to more efficient induction of immune responses in the airway mucosa, which is critical to control or prevent the SARS-CoV-2 infection.

The emergence of the delta variant, which became of high epidemiological relevance given its marked pathogenicity and transmissibility and is likely to evade the immunity induced by the Wuhan strain [43], prompted us to design a new version of the LTB-RBD carrying the delta-specific sequence. The expression of this new antigen led to its recovery as an insoluble protein with similar yields to those observed for LTB-RBD Wuhan. Given the regulatory issues associated with using the His tag, this new construct lacks tags, and the purification strategy was then established to account for a tag-free procedure. Anionic exchange chromatography allowed purifying the LTB-RBD-delta antigen to homogeneity. The previously standardized conditions for refolding of the LTB-RBD Wuhan allowed us to successfully refold the LTB-RBD delta antigen with similar yields with respect to the former, suggesting that the methods developed are robust and could be easily applied to newer variants. We are currently assessing this approach to produce an LTB-RBD omicron antigen.

## 5. Conclusions

The distinct versions of the LTB-RBD antigen obtained in this study constitute promising candidates for developing vaccines for which detailed expression and purification protocols have been developed. The LTB-RBD production platform used offers low cost, absence of tags, and easy adaptation to new variants, while supporting the development of mucosal vaccines. The obtained LTB-RBD antigens generate the perspective to achieve mucosal anti-COVID-19 vaccines, which promise to induce sterilizing immunity against SARS-CoV-2.

## Figures and Tables

**Table 1:** Protein concentration after a third step of refolding buffer.

Second Refolding Step				
Formulation	50 mM Na_2_CO_3_, 10% (*v*/*v*) Glycerol, 0.01% (*v*/*v*) Tween 20, pH 9.2			
Protein concentration (μg/mL)	18.23			
Protein recovery	100%			
**Third Refolding Step**				
Formulation	PBS 1×, 0.01% (*v*/*v*) Tween 20	PBS 1×	10 mM Phosphate, 9% (*w*/*v*) sucrose, 0.01% (*v*/*v*) Tween 20, pH 7.0	10% (*w*/*v*) Sucrose, 0.01% (*v*/*v*) Tween 20
Protein concentration (μg/mL)	4.57	0	2.09	15.30
Protein recovery (%)	25	0	11	87

## Data Availability

The data presented in this study are available on request from the corresponding author.
